# Darwin review: the evolution of virulence in human pathogens

**DOI:** 10.1098/rspb.2023.2043

**Published:** 2024-02-07

**Authors:** Sunetra Gupta

**Affiliations:** Department of Biology, University of Oxford, Oxford, UK

**Keywords:** virulence, evolution, immunity, antigenic diversity, vaccination, public health

## Abstract

By definition, all pathogens cause some level of harm to their hosts. If this harm occurs while the pathogen is transmitting, it can negatively affect the pathogen's fitness by shortening the duration over which transmission can occur. However, many of the factors that increase virulence (i.e. harm to host) also promote transmission, driving the pathogen population towards an optimal state of intermediate virulence. A wider spectrum of virulence may be maintained among pathogen populations which are structured into multiple discrete strains though direct resource and immune-mediated competition. These various evolutionary outcomes, and the effects of medical and public health interventions, are best understood within a framework that recognizes the complex relationship between transmission and virulence in the context of the antigenic diversity of the pathogen population.

## 1. Introduction

Infectious diseases remain a leading cause of human death in many parts of the world, with lower respiratory infections ranking higher than ischaemic heart disease in low-income countries in the period 2000–2019. Diarrhoeal diseases, malaria, tuberculosis and HIV/AIDS follow in the list of the top ten leading causes of death in these areas [1].

An understanding of the cellular and molecular mechanisms precipitating severe clinical outcomes of infections can be augmented significantly by placing them in the context of the ecological and evolutionary processes that permit the more virulent forms of the pathogen to persist is spite of the obvious detriment to the host which also, perforce, curtails their transmission. The natural expectation here is that pathogen populations will evolve to be as benign as possible, collectively, to their host. That this does not always occur can be explained in evolutionary terms as a result of a trade-off between virulence and transmissibility as certain factors which maintain transmissibility may be inextricably linked to the propensity to cause undesirable clinical outcomes [2].

There are several ecological processes (e.g. density-dependent mortality) which determine the precise outcome of these tensions between processes affecting virulence and transmissibility [3]. In this review, I develop a framework based on the antigenic diversity of the pathogen population with the aim of demonstrating the critical role of acquired immunity to infection and disease in shaping the evolution of virulence, as well as defining the routes by which we can reduce the burden of disease in human populations.

## 2. A basic framework for pathogen evolution in the context of antigenic diversity

**Figure 1.**
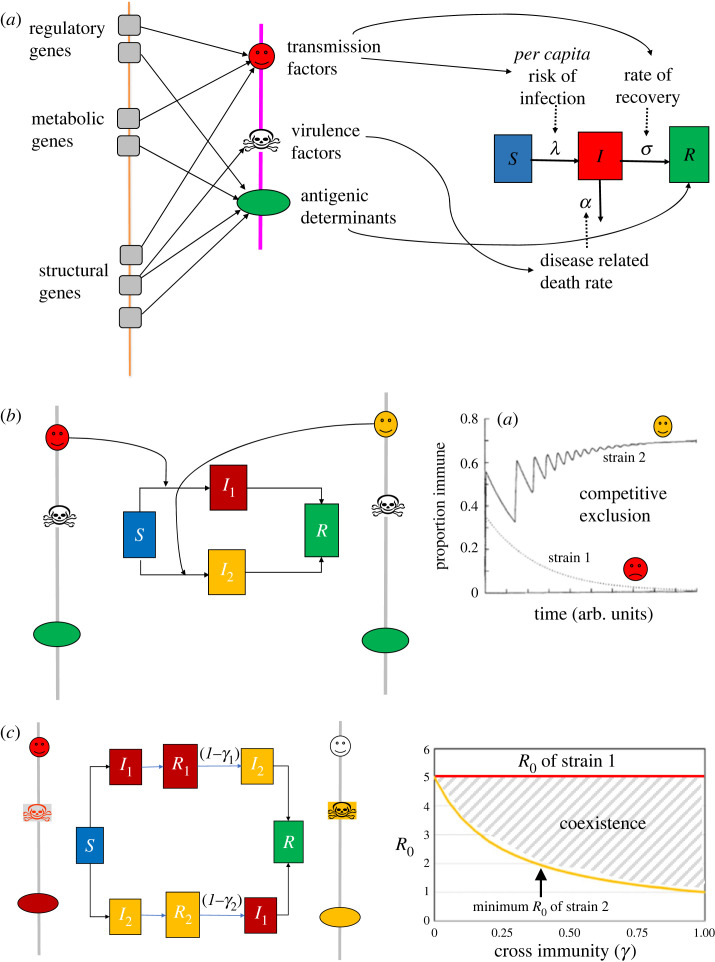
A conceptual framework for evolution of pathogen virulence. (*a*) Pathogen genes map onto features dictating transmissibility, virulence and antigenic determinants which influence specific parameters in a simple SIR model of infection dynamics (*λ* is the *per capita* risk of infection, *σ* the recovery rate from infection and *α* the disease-related death rate). (*b*) Under strong cross-immunity (invariance of antigenic determinants), the strain with the highest *R*_0_ wins. (*c*) Cross-immunity may be incomplete (0 < *γ*<1) and also asymmetric (*γ*_1_ > *γ*_2_) between strains, giving rise coexistence between strains [2] within certain bounds of difference in *R*_0_.

The evolution of virulence within a pathogen population has to be understood in the context of how its genotype maps onto its phenotype. We can conceptualize each individual pathogen as containing elements which determine its virulence and transmissibility, as well as its antigenic characteristics (figure 1*a*). These determine the components of theoretical frameworks that we employ to study the epidemiology and evolution of infectious disease systems.

For example, the classic S-I-R model [2], in which members of the host population are categorised as being susceptible, infected or recovered, assumes that the pathogen population is uniform across the transmission factors which govern the per capita risk of infection (*λ*) and recovery rate (*σ*), the virulence factors which determine the disease related death rate (*α*) and the antigenic determinants which elicit lifelong immunity.

A way to characterize the fundamental transmissibility of the pathogen is by its basic reproduction number, *R*_0_, which is equivalent to the average number of secondary cases generated by a primary infection in a totally susceptible population [2]. This is essentially the maximum transmissibility of the pathogen and can be expressed as a function of the relevant parameters: for example, within the system described in figure 1*a*, *R*_0_ = *β*/(*σ* + *α*), where *β* is a combination of parameters linearly relating the per capita risk of infection, *λ*, to the proportion infected. Model structures may be expanded to include demographic parameters, details of contact networks and other factors, but many basic behaviours are determined by the fundamental transmission potential, *R*_0_.

An important feature of this system is that it will tend towards an ‘endemic equilibrium’ in which the proportion of the population immune to infection hovers around 1–1/*R*_0_, regardless of how quickly immunity against infection is lost. In the case of pathogens which confer lifelong immunity upon infection, this occurs through the slow diminution through death of the immune population being balanced by their slow replenishment through infection of immune-naive newborns. For many other pathogens, such as SARS-CoV- 2, the same level of immunity in the host population at endemic equilibrium (i.e. of the order of 1–1/*R*_0_) is maintained by rapid loss of infection-blocking immunity and reinfection [4,5].

## 3. Evolution of virulence under complete cross-immunity

Differences in *R*_0_ will arise due to mutations in the genes that contribute to transmission and virulence. The model structure may be altered to accommodate such variation by differentiating between strains on the basis of these characteristics (figure 1*b*). A simple rule that emerges from these multi-strain models with no variation in antigenic determinants (i.e. perfect cross-immunity) is that the strain with the highest *R*_0_ eventually outcompetes all other strains [6,7].

A basic corollary here is that, contrary to popular belief, these systems will not always evolve towards low virulence. This is because the same genes which increase virulence, and thereby reduce *R*_0_, may also increase transmission. As a consequence, these systems will evolve towards an optimal virulence which maximizes *R*_0_. The canonical example of evolution towards an optimal intermediate virulence comes from our records following the release of the myxoma virus in Australia in 1950 to contain the rabbit population which was spiralling out of control. An extremely virulent (at least in European rabbits) form of the virus was released but was observed to attenuate over the years. Laboratory studies indicated that the virus could evolve to be even milder; but instead it settled to an intermediate level of virulence, most likely because the sores caused by these strains aided the mechanical transmission of the virus and thus gave them an advantage over even milder strains that did not induce these symptoms [8]. In the meantime, rabbits became more resistant, thereby reducing the transmission advantage of very mild strains. Similar evolutionary outcomes were observed in Europe following the release in France of a separate South American strain of myxoma virus in 1952; studies have shown that this convergence occurred at the level of phenotype rather than genotype, with a several different mutations in a constellation of genes producing similar changes in virulence [9]. This underscores the point that the optimization of virulence within a pathogen population is driven by the complex relationship between genotype and phenotype [10].

Pathways to acquisition and loss of virulence may, nonetheless, be tightly constrained as suggested by studies recording the mutations that lead to reversion to a virulent phenotype in live attenuated oral polio vaccines. It has been shown that polio viruses are able to replicate faster in cell culture when they acquire mutations which increase virulence (i.e. survival times after challenge) in a mouse model [11]; remarkably, these are the very same mutations that are associated with epidemics of vaccine-derived polio observed in Nigeria, Madagascar, Egypt, Belarus and China. The evolutionary trajectories available to a pathogen population are thus strongly determined by the set of viable options in both functional and competitive terms.

It should be mentioned here that the same principles operate for infectious disease systems within which there is no recovery from infection—in other words, an SI rather than an SIR system. This is because the dynamics of infectious diseases are primarily dictated by the proportion of the population available for infection which can be diminished by either long-lasting infection or acquired immunity. Thus, another putative example of evolution towards intermediate virulence is provided by observations regarding changes in viral loads of HIV-1 over time. The natural course of HIV-1 infections involves an extended period of stable viral load, often referred to as the ‘set-point’. During this period, CD4+ T cells are steadily destroyed at a rate proportional to the set-point viral load (SPVL), thereby creating a clear association with virulence. Fraser and colleagues conjectured that since the transmission rate of HIV-1 was also likely to increase with viral load, the virus population would evolve towards an optimal SPVL [12]. They were able to substantiate both these claims through a large longitudinal study in Uganda which showed a strong correlation between SPVL and likelihood of HIV transmission within sero-discordant couples, as well as a significant decline in SPVL over 20 years as predicted under these circumstances [13].

## 4. The role of immune evasion

Evolution towards a state of optimal virulence, in which R_0_ is typically maximized, occurs within systems where there is strong competition between variants, often mediated by cross-immunity as shown above. Conceptually, the latter corresponds to invariance in the principal antigenic determinants. Among pathogens where the principal targets of protective immunity are diverse, levels of cross-immunity between strains may be lower; this results in a relaxation of competition which then permits coexistence between strains with different R_0_ to a degree that is commensurate with the levels of cross immunity (*γ*) between them (figure 1*c*).

Competition between strains may also be relaxed if immunity develops in such a way as to permit co-infection: effectively, this reduces cross immunity (*γ*) and thereby can permit a degree of coexistence between strains of different virulence [14]. Co-infection may even reverse the outcome of competition between strains if the more virulent type is able to dislodge the less virulent type within the host, even though the latter is more successful in transmitting between hosts. Many such elaborations have been proposed for host-pathogen systems with strong cross-immunity [3,15] but they generally lead to the dominance of a single strain (with either lower or higher virulence than expected within a model without co-infection) or narrow band of strains with similar characteristics.

An interesting example of coexistence arising through evasion of immunity can be found in the *Bordetellae* bacterial species, two members of which (*B. pertussis* and *B. parapertussis*) cause whooping cough and co-circulate in human populations, having both evolved from *B. bronchoseptica*. *B. parapertussis* is believed to have emerged more recently, which would have required it to breach the barriers imposed through cross-immunity in the population arising from exposure to *B. pertussis*. Mouse studies [16] indicate that the basis of this immune evasion is the retention of the O antigen in *B. parapertussis;* this antigen is absent in *B. pertussis.* The O antigen is a component of the lipopolysaccharide (LPS) structures within the outer leaflet of gram-negative bacteria and has been implicated in various immune evasion processes, such as complement-mediated killing, and antimicrobial peptide-mediated bactericidal effects. In this specific instance, the O antigen of *B. parapertussis* was shown to inhibit binding of cross-reactive antibodies in mice previously immunised with *B. pertussis* thereby permitting colonization. This implies, of course, that cross-immunity between these pathogens is asymmetric (*γ*_1_ > *γ*_2_ in figure 1*c*) as *B. pertussis* would not be able to colonize a host already immune to *B. parapertussis*. Because of this, *B. parapertussis* can theoretically outcompete *B. pertussis* even if *B. parapertussis* is of lower transmissibility (*R*_0_), and coexistence can thus only occur if this condition is met [17]. With regard to virulence, it is believed that *B. parapertussis* produces milder symptoms than *B. pertussis*, thus demonstrating that coexistence between similar pathogens or different strains of the same pathogen allows variation in clinical phenotype.

## 5. Emergence of discrete multi-locus strain types

**Figure 2.**
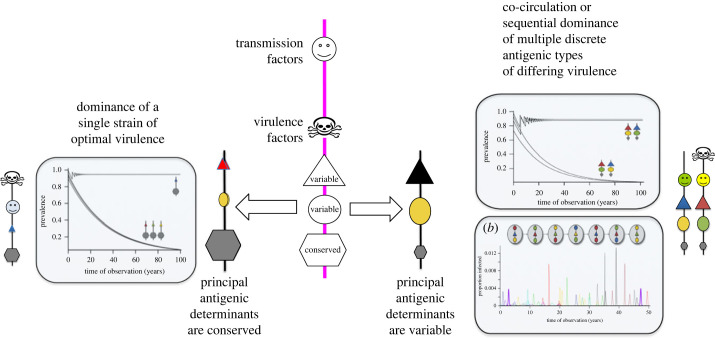
The spectrum of virulence within a pathogen population is determined by the variability of its principal antigenic determinants. All pathogens contain antigenic determinants which are conserved, as well as some that have the capacity to vary without loss of pathogen function. In the case where the principal antigenic determinants are conserved, there is strong competition between all strains and this leads to the dominance of a single strain of optimal virulence. By contrast, when the principal antigenic determinants are variable, we observe either the stable co-circulation or sequential dominance of discrete antigenic types [18] which may vary in virulence.

We thus arrive at a set of evolutionary outcomes according to the level of diversity in the dominant antigenic determinants within a pathogen population (figure 2). When the dominant determinants are conserved (e.g. for measles) we observe evolution towards a single state of optimal virulence whereas if they are variable, we may expect variation in virulence among the co-circulating discrete strains [6]. It should be noted here that, within a multi-locus framework, discrete strain structure arises due to the polarisation of strains in a complex antigenic space under strong immune selection [18–21], in spite of high levels of mutation and/or genetic exchange [22]. Many bacterial pathogens (pneumococcus, meningococcus, Group A streptococcus) serve as examples of where such discrete strain structure persists over long periods, as do populations of the malaria parasite, *Plasmodium falciparum*. In these infectious disease systems, there can be considerable variation both in severity and other fundamental clinical characteristics among the constituent strains.

In *P. falciparum*, a very neat connection exists between the clinical characteristics of a strain and its antigenic type as both are linked to the highly variable antigen PfEMP1 which is expressed on the surface of an infected red blood cell by the parasite for the purpose of mediating adhesion to different tissues. Different variants of PfEMP1 thus cause different symptoms depending on their predilection for certain receptors: those that cause cerebral malaria, for example, preferentially stick to ICAM-1 which is abundant in the brain, while pregnancy-associated malaria is caused by a form which sticks to placental chondroitin sulfate [23]. PfEMP1 is also a primary target of protective antibody responses which are highly specific to each variant. Thus, the forces of immune selection that partition the population into discrete strains may also directly maintain a spectrum of virulence among malaria parasites.

Where the virulence characteristics are not embedded within the antigenic components of a pathogen, it is harder to see how differences in virulence would be maintained in the face of genetic exchange or high mutation rates. To answer this question, it is important to recognize that pathogens also compete directly for resources such as nutrients and host receptors, and this can cause the population to self-organise into discrete metabolic types. To minimize both direct resource competition and indirect immune competition, we may expect non-overlapping associations to arise between antigenic and metabolic types as well as with virulence factors [24]. This would explain the high level of linkage disequilibrium observed across the genomes of many bacterial pathogen populations which were previously ascribed to lack of genetic exchange. Alternatively, immune selection may cause the stable diversification of a critical element which interacts with many genes: using machine learning techniques to analyse whole genome data, we have demonstrated that the lineage structure of the pneumococcus may be driven by immune selection on the groEL heat-shock protein [25]. Fundamentally, a combination of immune selection in conjunction with direct resource competition and/or epistasis can generate stable associations between virulence factors and antigenic types. The strong associations between serotypes of many bacterial pathogens and propensity to cause invasive disease can be more parsimoniously explained due to these selective processes rather than having arisen through neutral processes.

## 6. Unstable strain dynamics

The multi-locus framework which has been used to develop the arguments in this review can also be employed to understand the evolution of virulence among pathogen populations that are characterized by the sequential appearance of strains rather than their stable coexistence or the dominance of a single strain [18].

If a new variant arises in a pathogen population, it may quickly outcompete one which is currently circulating in the population provided it has even a small advantage either due to increased *R*_0_ or because it is sufficiently immunologically different. While the virulence of a variant will impact upon its *R*_0_, this does not mean that successive variants will necessarily become milder for the same reasons outlined above, namely (i) lower virulence does always lead to higher transmissibility, and (ii) immune evasion can compensate for a decline in R_0_.

Eventually, the pathogen population may converge to a single optimal state or conversely, remain in a perpetual state of instability, exhibiting sequential dominance of single or multiple strains. This state of cyclical or chaotic strain structure readily arises within multi-locus models at intermediate values of immunological cross-protection between strains that share variants [18], and may underpin the dynamics of a number of important human pathogens such an influenza [26].

Once again, if the antigenic determinants are themselves virulence factors then we may expect the intrinsic propensity of the pathogen to cause harm to fluctuate over time. This has not been observed in human influenza to any measurable extent but it is evident that certain avian influenza strains periodically exhibit high virulence. The severe consequences of their occasional spread to humans, as well as the large toll they take on the poultry industry, make this an important public health and veterinary problem. Within the framework of multi-locus pathogen evolution, the emergence of highly pathogenic strains of avian influenza would require that certain antigenic types increase in transmissibility when they acquire the polybasic cleavage sites (which are separate from the antigenic determinants) which confer increased pathogenicity. For other antigenic types, their lower pathogenicity counterparts are more successful and thus prevail when that antigenic type is favoured by the shifting landscape of host immunity. Factoring in host lifespan allows us to conclude that highly pathogenic strains may be expected to emerge regularly in short-lived birds but less frequently in longer-lived species [27]. In these circumstances, contact between species of different lifespans can provoke the emergence of a previously suppressed strain of high pathogenicity. To prevent such outcomes, it may be prudent to restrict farming practices which bring avian species into more prolonged contact.

Another route for a change in virulence to occur in human influenza is through the process of ‘antigenic shift’, whereby a new lineage invades, sometimes replacing currently circulating subtypes. Since these lineages are generated through reassortment of avian, swine and human influenza genes (with pigs typically acting as a ‘mixing vessel’), it is difficult to predict what changes in intrinsic virulence may accompany the event of a new influenza pandemic. However, whether or not a new strain or subtype will cause many deaths depends not only on its intrinsic virulence but also upon the immune status of the host population, as explained in the following section.

## 7. The role of immunity to severe disease

A crucial feature of pathogens which repeatedly infect their hosts is that the likelihood of developing severe disease upon reinfection is highly reduced from the infection fatality rates experienced by a naive individual. This is true of many infectious disease agents, such as *P. falciparum* malaria [28], where the first infection appears to confer a significant level of protection against severe disease. A simple extension of this argument allows us to provide a solution to the conundrum of why the 1918 influenza pandemic claimed so many lives, while none of the other lineage replacement events since then have taken such a toll. We can (quite reasonably) postulate that, prior to 1918, influenza would die out after each pandemic, whereas we now live in an era where the demographic characteristics and global movements of the human population prevent the extinction of the virus population. If this is correct, then the influenza virus of 1918 would essentially have entered a population in which the majority of those under the age of 30 years had no previous exposure to any form of influenza (since the last pandemic occurred in the early 1890s) and this could account for its particularly large death toll observed within that age class.

Immunity to severe disease may derive from exposure to other strains of the pathogen or even to related pathogen species. Several studies indicate that we may have enjoyed protection against severe disease from SARS-CoV-2 due to pre-existing B-cell and T-cell responses against related endemic coronaviruses [29,30]; within this context, the increase in infection fatality rate with age may be explained by assuming that the breadth of cross-protection contracts with age due to focusing of the immune response onto seasonal viruses through repeated exposure [31], although the primary driver in the oldest age groups was more likely to be immunosenescence [32].

Severity of symptoms may also increase with age for physiological reasons, as it does with diseases such as polio and chicken pox. A more indirect way in which age can affect the outcome of infection is when a pathogen which normally causes mild infection is nonetheless harmful to the fetus. The introduction of such a pathogen into a susceptible adult population can then produce a spate of birth defects, as we saw with the introduction of the Zika virus in South America which resulted in many infants being born with microcephaly. Rubella, which is also caused by a generally fairly innocuous virus, can cause a range of problems in the unborn child such as deafness, eye abnormalities and heart disease. The introduction of rubella to population with no immunity to it would be accompanied by an increase in congenital rubella syndrome. For both of these viruses, congenital defects become rare once they become endemic since most pregnant women are immune. Vaccination can guarantee that women of childbearing age will not contract rubella while pregnant but high coverage is required so as not to paradoxically increase rates of CRS by lowering the risk of infection, and thereby increasing the age at first infection, in the unvaccinated population.

Another reason that endemic diseases exhibit lower virulence is that infants are born with passively transferred maternal antibodies which often gives them protection against severe disease [33]; this provides a ‘window of opportunity’ for the infant to acquire lifelong immunity against severe disease during primary infection without the risk of adverse effects. Delaying the average age of first infection beyond this period through interventions can thus also appear to increase the virulence of the pathogen.

Fundamentally, the outcome of infection is not just determined by the intrinsic virulence of the variant but also by the profile of pre-existing immunity within an individual. A new variant will inevitably appear to be less virulent if it arrives within a population which has already encountered another variant of the same pathogen or possesses a significant level of protection against severe disease due to previous exposure to another related species. This caution must also be extended is assessing the virulence of a new variant such as SARS-CoV-2 omicron, which is widely assumed to be intrinsically milder than its predecessors simply because it did not cause as much hospitalisable illness and death. Recent studies (e.g. [34]) suggest that, in fact, it continued to carry the same dangers to vulnerable individuals.

Finally, resistance against disease may evolve within the host population on a longer timescale due to the selective advantage enjoyed by individuals who are at lower risk of dying due to heritable traits. There are several human polymorphisms that protect against severe manifestations of *P. falciparum* malaria, the most well-known of which is the sickle-cell disorder of haemoglobin. It has been proposed that the prevalence of sickle-cell trait can influence the genetic structure of *P. falciparum* populations [35]; while this could lead to coevolutionary cycling, the time scale of such changes within human populations may be of little public health relevance. Nonetheless, these processes could explain the periodic emergence of zoonotic pathogens from shorter-lived species whose resistance patterns may fluctuate more frequently under coevolutionary selection pressures.

## 8. Effects of pharmaceutical interventions

In considering the effects of pharmaceutical interventions on the evolution of virulence, it is important to distinguish between those which prevent infection and those which prevent disease. Vaccines which induce lifelong immunity against infection are unlikely to alter the intrinsic virulence of a pathogen as these mostly target conserved determinants which the pathogen cannot afford to alter. However, they may have perverse consequences by altering the average age of infection, such as in the rubella example discussed above; these may be avoided by keeping rates of vaccination high.

An alternative strategy for pathogens which elude the development of infection-blocking vaccines is to aim to reduce disease severity: vaccines against SARS-CoV-2 are an example of the latter. In theory, this could cause a shift towards higher virulence since the survival of the host could effectively increase the *R*_0_ of more virulent variants and thereby allow them to outcompete milder variants [36]. The observed increase in virulence of Marek's disease among poultry following mass vaccination has been attributed to this effect. The recent recovery of Marek's disease virus (MDV) from skeletons of archaeological chickens [37] confirm that the virus has been circulating for over 1000 years, and attest to a significant increase in virulence since that time.

Drugs and vaccines which prevent disease without affecting transmission can also impede the journey of a pathogen population towards a lower optimal virulence. The widespread use of combination therapy could thus reconcile the recent identification of a highly virulent variant (VB) of HIV-1 in the Netherlands [38] in contrast with the findings of evolution towards lower viral loads in the Uganda study described above. Phylogenetic studies indicate that the VB variant arose in the 1990s, well before successful treatment was in place, thereby emphasizing that the evolution of virulence tends to occur through selection of pre-existing variants rather than provoking new mutations.

Among pathogen populations that are stably structured into strains, the effects of vaccination may be more complex. Targeting conserved determinants is naturally the preferred route for vaccination but, because these are perforce not naturally immunogenic, this is a difficult task. Nonetheless, important strides have been made in this direction which could lead to superior control of diseases such as malaria [39]. Another option is to target only the virulent strains if they are easily identifiable, as is the case with *Streptococcus pneumoniae* for which our current vaccination strategy focuses only on those antigenic variants which are associated with invasive disease. However, this can lead an unwanted evolutionary outcome whereby previously non-virulent strains acquire the same characteristics as the disease-causing strains against which the vaccine has been developed [40,41]. It is important that we develop strategies (such as regular recycling of strain-specific vaccines) informed by evolutionary theory to prevent such perverse consequences.

## 9. Conclusion

The toll taken by infectious diseases in human populations can be reduced by designing strategies which embed an understanding of the evolutionary dynamics of the relevant pathogen populations in their epidemic and endemic phases, with particular reference to the trajectory of virulence.

The simplistic notion that all pathogens evolve towards low virulence has long been replaced with the more nuanced view that pathogen populations stagger towards an ‘optimum’ virulence which maximizes transmissibility. This is the consequence of strong competition between variants differing in their virulence and transmissibility, typically mediated by immunity against shared conserved antigenic determinants. The precise outcome of this intense competition is determined by many factors (such as possibility of co-infection) such that the simple rule that *R*_0_ itself is always maximized does not always hold [3], but these systems are typically characterized by the dominance of a unique strain or a very small subset of variants. Due to the existence of naturally protective conserved antigenic determinants, it has been relatively straightforward to develop vaccines against many pathogens in this category (e.g. measles) and the successful control of disease is best effected by wide uptake of such vaccines.

In the case of pathogens which lack such naturally protective conserved antigenic determinants, vaccines have been harder to develop. These systems tend to self-organize into discrete strains, both at an antigenic and metabolic level, such that competition between co-circulating strains is minimized. As a result, they can support a high level of variance in virulence between strains. Reducing the disease burden within these systems is a major public health challenge but can be effected by targeting those of high virulence, although this may cause rearrangements within the pathogen population that negate the initial benefits. A crucial feature of many of these infectious disease systems (e.g. malaria, SARS-CoV-2) which is only now starting to be exploited as a method of reducing mortality is that immunity against severe disease is often established upon first or second infection, even though the host remains open to repeated infection. Vaccines against these diseases can therefore be directed towards protecting against severe disease rather than infection.

Placing our understanding of the evolution of virulence within the context of naturally acquired and vaccine-derived immunity to pathogens provides us with a robust framework for predicting the changes in the ability of a pathogen to cause disease, as well as informing us of the choices we can make to reduce their burden in human populations.

## Data Availability

This article has no additional data.
